# Outcomes of metastatic spinal cord compression secondary to primary hepatocellular carcinoma with multidisciplinary treatments

**DOI:** 10.18632/oncotarget.15708

**Published:** 2017-02-25

**Authors:** Shaohui He, Haifeng Wei, Yifei Ma, Jian Zhao, Wei Xu, Jianru Xiao

**Affiliations:** ^1^ Department of Orthopedic Oncology, Spinal Tumor Center, Changzheng Hospital, Second Military Medical University, Shanghai, China

**Keywords:** metastatic spinal cord compression, hepatocellular carcinoma, multidisciplinary treatments, progression-free survival

## Abstract

Few studies have reported progression-free survival (PFS) and overall survival (OS) of Metastatic spinal cord compression (MSCC) patients with primary hepatocellular carcinoma (HCC) following surgery and adjuvant therapies. Enrolled in this study were 155 MSCC patients with primary HCC who received surgery and adjuvant therapies between 2000 and 2015. Kaplan-Meier methods and Cox’s proportional hazards regression mode were performed to investigate the clinical features and prognostic factors affecting PFS and OS. The median PFS and OS was 7.0 months and 9.7 months, respectively. 92.9% patients responded well to surgery according to the Visual Analogue Scale, Frankel Score and postoperative complication occurrences. 68 (43.9%) patients who received circumferential decompression achieved better PFS than the remaining 87 (56.1%) patients who received laminectomy. Favorable outcomes were achieved after surgery during the perioperative period. Circumferential decompression was associated with better PFS than laminectomy. The postoperative Frankel Score E, Eastern Cooperative Oncology Group performance score of 1 or 2, no visceral metastasis, administration of postoperative radiation and the use of Sorafenib were found to be significant predictors of better PFS and OS. Patients who previously underwent resection of primary HCC with/without liver transplantation tended to have a better OS.

## INTRODUCTION

Hepatocellular carcinoma (HCC) is the most common pathological type of liver cancer, with an incidence of fifth in men and seventh in women worldwide. Globally, it is the second most frequent cause of cancer-related deaths in men and the sixth in women [1–4]. The morbidity and mortality rates of HCC are especially high in East-Asia and Sub-Saharan Africa [5]. HCC is associated with a high rate of chronic hepatitis B virus (HBV) or hepatitis C virus (HCV) infection [6–8]. Bone is the second most common metastatic site of HCC [9, 10], accounting for 20% of all cases [7, 11, 12]. The axial skeleton is the most frequent location of bone metastasis, which is probably correlated with the high portal hypertension caused by advanced cancer [13, 14].

The incidence of metastatic spinal cord compression (MSCC) caused by primary HCC has been encountered more frequently in recent years due to improved diagnosis and therapeutic modalities of primary HCC. Severe pain and neurological deficits such as bower and bladder incontinence and loss of ambulation in MSCC patients from primary HCC often lead to an unsatisfactory quality of life [15, 16]. Surgery remains the treatment of choice to achieve immediate decompression, stabilization of the axial skeleton and improvements of outcome [17, 18], even though spinal metastasis of HCC is recognized as a terminal stage of disease, for which standard treatments of corticosteroids and radiotherapy are generally recommended [19, 20]. To the best of our knowledge, few studies have reported the outcomes and relevant factors of clinical features affecting PFS and OS in a large sample of patients with primary HCC-related MSCC who received surgery and adjuvant therapies. Consequently, we made a comprehensive review of a cohort of patients, hopefully to provide a relatively credible reference for evidence-based decision-making in the treatment of primary HCC-related MSCCs.

## MATERIALS AND METHODS

### Patients and follow-up strategy

Patients who were diagnosed with MSCC from liver cancer and received treatment in our institution between January 2000 and December 2015 were reviewed retrospectively for potential eligibility. After excluding patients who received non-surgical therapies, and those who received surgery merely for pathological biopsy and those who were histologically confirmed to have cholangiocarcinoma, a total of 155 patients with primary HCC were enrolled in this study. The surgical strategies were mainly based on the revised Tokuhashi Scoring system [21] and the Tomita Scoring system [22].

Informed consent was obtained from all patients involved. The study protocol was approved by the Institutional Review Board of Changzheng Hospital prior to initiation of the study and conducted according to the guidelines approved by the ethics committee. The clinical and operative records, image data, blood tests results and pathological reports of the patients were reviewed by two independent researchers. Positron emission tomography-computed tomography (PET-CT) was done to find possible metastatic sites. Child-Pugh Grade, pre- and postoperative Frankel Score (evaluated between 1-3 months after surgery) and the Eastern Cooperative Oncology Group performance score (ECOG-PS) [23] were used to evaluate the liver function, neurological status and performance status.

Surgical treatment was performed within three days after diagnosis of MSCC. The surgical approaches utilized were based on the revised Tokuhashi Scoring system [21] and the Tomita Scoring system [22], consisting of circumferential decompression (including en-bloc) and laminectomy decompression. Cisplatin was used intraoperatively for local chemotherapy in cases where the dura was unbroken. Systemic chemotherapy [not including trans-catheter arterial chemotherapy and embolization (TACE) or Sorafenib], radiotherapy, bisphosphonate (zoledronic acid) were administered to help prevent skeletal related events. And Sorafenib was selected on the basis of personalized evaluation.

All patients were followed up monthly for the first three months and at 3-month intervals for the next twelve months on an outpatient basis. The patients’ clinical condition and radiographic findings were obtained to evaluate the prognosis. Patients data collection in the present study was supported by the National Cancer Register Centre and Shanghai Municipal Bureau of Public Security.

### Statistical analysis

Progression-free survival (PFS) was the primary endpoint and defined as the time period between the date of surgery and the date of deterioration of the patients’ neurological function or progression (either primary or metastatic lesion) based on radiological assessments. Overall survival (OS) was a key secondary endpoint and defined as being from the first day after surgery until the date of the patients’ death due to disease or the end of December 2015. Quantitative data were described by mean or median (range), and qualitative data by counts and percentages. The Kaplan-Meier curve was adopted to estimate the cumulative survival rate, with log-rank test to identify the difference. Variables with *p* value≤ 0.10 were subjected to multivariate analysis using the Cox proportional hazards model. All statistical analyses were performed using SPSS statistics, version 21.0 (IBM corp., New York, USA), with *p* value of less than 0.05 being considered statistically significant.

## RESULTS

### Statistical description of the clinical features

The median age of the patients in this study was 50 (Range 29-79) years, with 119 (76.8%) males and 36 (23.2%) females. The HBV infection rate was 58.1% (90/155). Metastatic lesions were located in the cervical spine in 28 (18.1%) patients, thoracic spine in 69 (44.5%), lumbar spine in 53 (34.2%), and sacrum in 5 (3.2%). Mono-centric and multifocal lesions involving discontinuous vertebral bodies occurred in 112 (72.3%) and 43 (27.7%) patients, respectively. The preoperative duration of symptoms ranged from 0.5 to 12.0 months, with a median of 4.3 months. The median PFS and OS of the MSCC patients was 7.0 (Range 0.5-59.0) months and 9.7 (Range 1.0-59.0) months, respectively. According to the Kaplan-Meier curve, the 1-year PFS and OS rates were 31.1%, 51.9%, respectively. Notably, 75 (48.4%) patients had surgical resection of their primary lesions with or without liver transplantation before admission to our department. Details of the therapeutic protocols and outcomes of patients are illustrated in Table 1.

**Table 1:** Therapeutic protocols and outcomes of 155 MSCCs from primary HCC

Treatments	Progression-free survival		Overall survival	
	Alive	Died	Alive	Died
SR+CT+RT+Sora.+BS	9	8	9	8
SR+CT+RT+Sora.	2	9	1	10
SR+CT+RT+BS	10	6	9	15
SR+RT+Sora.+BS	3	14	3	6
SR+CT+Sora.+BS	2	0	1	1
SR+CT+Sora.	3	3	0	6
SR+CT+RT	8	1	2	16
SR+CT+BS	6	0	2	12
SR+RT+BS	3	8	5	6
SR+RT+Sora.	0	10	0	1
SR+Sora.+BS	0	8	0	0
SR+CT	9	0	2	24
SR+RT	2	7	2	7
SR+Sora.	0	17	0	0
SR+BS	1	3	0	4
SR	0	3	1	2
Total	58(5.6M*)	97(6.7M*)	37(8.0M*)	118(10.2M*)

SR: surgical resection; CT: chemotherapy; RT: radiotherapy; Sora: Sorofenib; BS: bisphosphonate

*: These four figures were medians of follow-up and survival time.

### Assessment of perioperative outcomes

The most common complaints included nocturnal back pain, extremity numbness and paraplegia. The mean preoperative Visual Analogue Scale (VAS) was 7.32 (median7.11, range 1-10), and Frankel score ranged from A to D. No patient died during the perioperative period. The mean postoperative VAS was 4.12 (median 4.08, range 1-7). The postoperative Frankel score ranged from C to E. Eleven (7.10%) patients had delayed wound healing. All healed after strengthening antibiotics, nutritional support (nine cases) and wound debridements (two cases).

### Statistical analysis of potential independent factors

**Figure 1:**
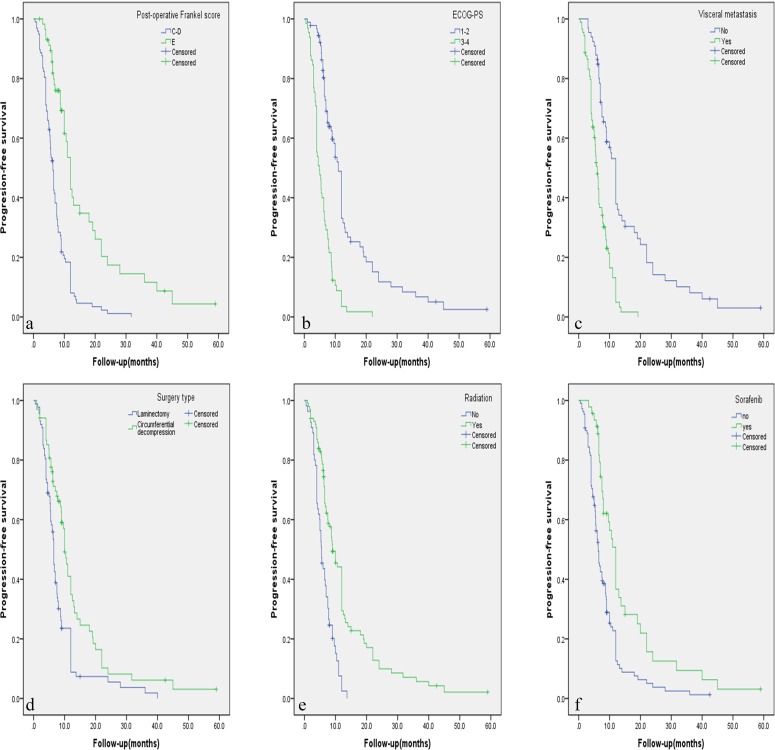
**A**.-**E**. Kaplan-Meier curves of progression-free survival based on six independent factors for prognosis.

**Figure 2:**
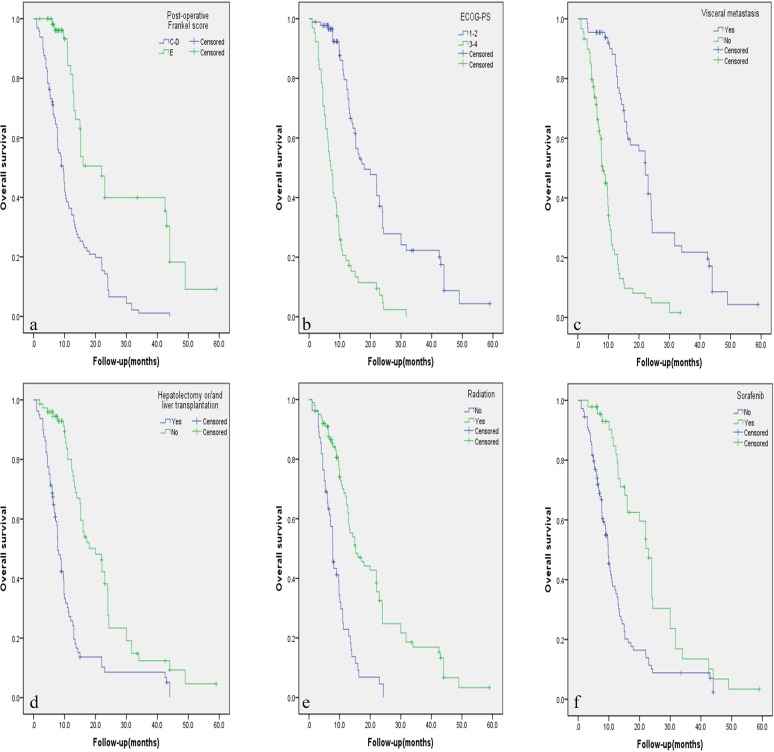
**A**.-**F**. Kaplan-Meier curves of overall survival based on six independent factors for prognosis.

The results of univariate prognostic analysis are demonstrated in Table 2. Based on the inclusion criteria of *p*≤0.10, eleven potential factors were submitted into the Cox regression model (Table 3). Patients with visceral metastasis had poorer prognosis with *p* = 0.011 for PFS (Hazard Ratio [HR] = 1.743, confidence interval [CI] = 1.135-2.677), and *p* < 0.001 for OS (HR = 2.759, CI = 1.686-4.515). Postoperative Frankel Score of E was a strong predictive indicator for better PFS (HR = 0.523, CI = 0.331-0.828, *p* = 0.006) and OS (HR = 0.453, CI = 0.271-0.758, *p* = 0.003). ECOG score of 3 or 4 was recognized as a poor predictor with *p* = 0.006 for PFS (HR = 1.832, CI = 1.187-1.827), and *p* = 0.035 for OS (HR = 1.697, CI = 1.038-2.773). Postoperative radiation was a beneficial indicator for PFS (HR = 0.606, CI = 0.406-0.906, *p* = 0.015) and OS (HR = 0.433, CI = 0.279-0.670, *p* < 0.001). Sorafenib was a favorable factor for PFS (HR = 0.583, CI = 0.363-0.984, *p* = 0.006) and OS (HR = 0.425, CI = 0.236-0.766, *p* = 0.004). Patients who previously underwent hepatecotomy and/or liver transplantation tended to have better OS (HR = 0.517, CI = 0.322-0.829, *p* = 0.006). Circumferential decompression was associated with better PFS than laminectomy (HR = 0.637, CI = 0.443-0.917, *p* = 0.015), but not for OS (*p* = 0.551). Independent factors affecting PFS and OS rate are illustrated in Figure 1 and Figure 2 respectively by using the Kaplan-Meier Method.

**Table 2:** Results of univariate analysis of clinical features

Factor	No. of patients	Progression-free survival		Overall survival	
		%	*P* value	%	*P* value
Age					
<50ys/>=50ys	75/80	12.0/17.5	0.449	20.0/32.5	0.257
<60ys/>=60ys	127/28	14.2/17.9	0.144	24.4/35.7	0.561
Gender: male/female	119/36	16.8/8.3	0.311	27.7/22.2	0.447
Mono/multifocal lesion	112/43	16.3/14.3	0.069	29.5/18.6	<0.001
Lesion location: C/T/L/S	28/69/53/5	21.4/14.5 /11.3/20.0	0.788	46.4/21.7 /20.8/40.0	0.879
Duration of symptom:<6M/>=6M	97/58	15.5/13.8	0.133	27.8/24.1	0.379
Visceral metastasis: no/yes	89/66	18.2/12.4	<0.001	33.3/21.3	<0.001
Other bone metastasis: no/yes	115/40	17.4/7.5	0.156	28.7/20.0	0.738
Frankel score					
preop: D/A-C	71/84	16.7/12.7	0.126	28.6/23.9	0.325
postop: E/C-D	58/97	32.8/4.1	<0.001	58.6/7.2	<0.001
ECOG-PS:1-2/3-4	90/65	24.4/1.5	<0.001	41.1/6.2	<0.001
Year:2000-2010/2010-2015	43/112	14.3/16.3	0.117	25.0/30.2	0.231
Spinal canal stenosis:no/yes	46/109	14.7/15.2	0.443	25.7/28.3	0.438
Paravetebral soft tissue neoplasm: no/yes	97/58	17.5/10.3	0.192	29.9/20.7	0.200
weight loss:<5kg/>=5kg	114/41	17.5/7.3	0.178	30.7/14.6	0.187
TACE: no/yes	95/60	10.5/21.7	0.082	17.9/40.0	0.073
Hepatecotomy or liver tranplantation: no/yes	80/75	7.5/22.7	<0.001	15.0/38.7	<0.001
Child-pugh grade: A/B-C	53/102	17.0/13.7	0.225	28.4/22.6	0.730
HBsAg: negative/positive	65/90	16.9/13.3	0.296	27.7/25.6	0.336
AFP:<20U/20-400U/>400U	46/65/44	17.4/18.5 /6.8	<0.001	30.4/30.8 /15.9	<0.001
Surgery type: lamilectomy/ circumferential decompression	87/68	9.2/22.1	<0.001	17.2/38.2	0.082
Surgery time					
<2h/>=2h	26/129	23.1/13.2	0.831	30.8/25.6	0.406
<3h/>=3h	94/61	13.8/16.4	0.433	25.5/27.9	0.376
Blood loss					
<2000/>=2000	87/68	19.5/8.8	0.192	29.9/22.1	0.322
<2500/>=2500	121/34	17.4/5.9	0.653	28.9/17.6	0.840
Chemotherapy: no/yes	37/118	10.8/16.1	0.628	32.4/24.6	0.448
Radiation: no/yes	55/100	7.3/19.0	<0.001	12.7/34.0	<0.001
Sorafenib: no/yes	109/46	11.9/21.7	<0.001	23.9/32.6	<0.001
Bisphosphonate: no/yes	75/80	4.1/24.1	<0.001	13.5/38.3	0.001

**Table 3:** Results of multivariate analysis of potential prognostic factors

Factors	Progression-free survival		Overall survival	
	HR (95%CI)	*P* value	HR (95%CI)	*P* value
Mono/multifocal lesion	-	0.057	-	0.108
Visceral metastasis	1.743(1.135-2.677)	0.011	2.759(1.686-4.515)	<0.001
Postoperative Frankel score	0.523(0.331-0.828)	0.006	0.453(0.271-0.758)	0.003
Surgery type	0.637(0.443-0.917)	0.015	-	0.551
Hepatecotomy or liver tranplantation	-	0.681	0.517(0.322-0.829)	0.006
TACE	-	0.859	-	0.592
ECOG-PS	1.832(1.187-2.827)	0.006	1.697(1.038-2.773)	0.035
AFP	-	0.191	-	0.185
Radiation	0.606(0.406-0.906)	0.015	0.433(0.279-0.670)	<0.001
Sorafenib	0.583(0.363-0.934)	0.006	0.583(0.365-0.932)	0.024
Bisphosphonate	-	0.359	-	0.484

## DISCUSSION

**Figure 3:**
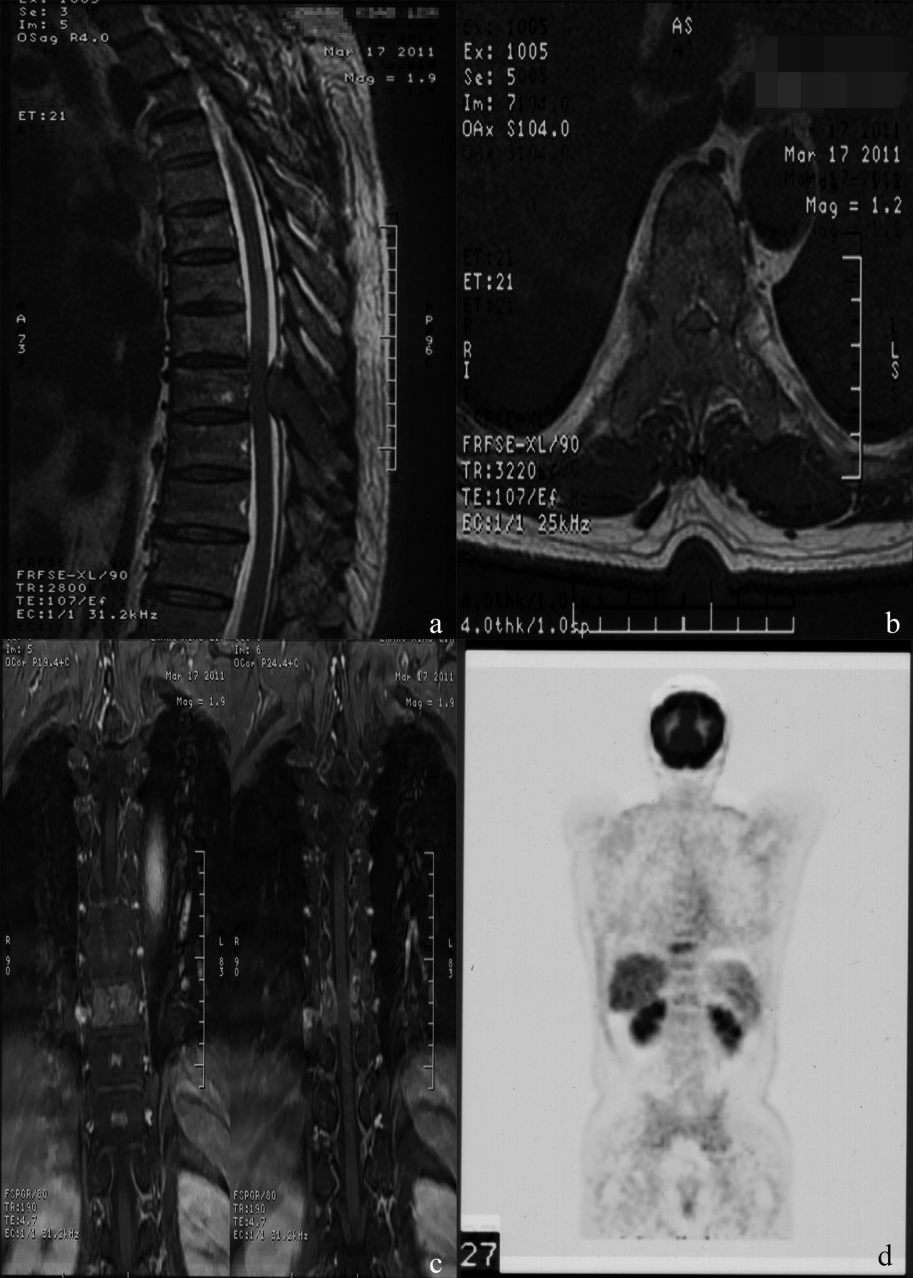
**A**.-**D**. Typical MRI and PET-CT images, showing abnormal signal of T9 body and appendix with spinal canal stenosis and spinal cord compression, abnormal ^18^F-fluorocholine uptake of the thoracic vertebrae.

**Figure 4:**
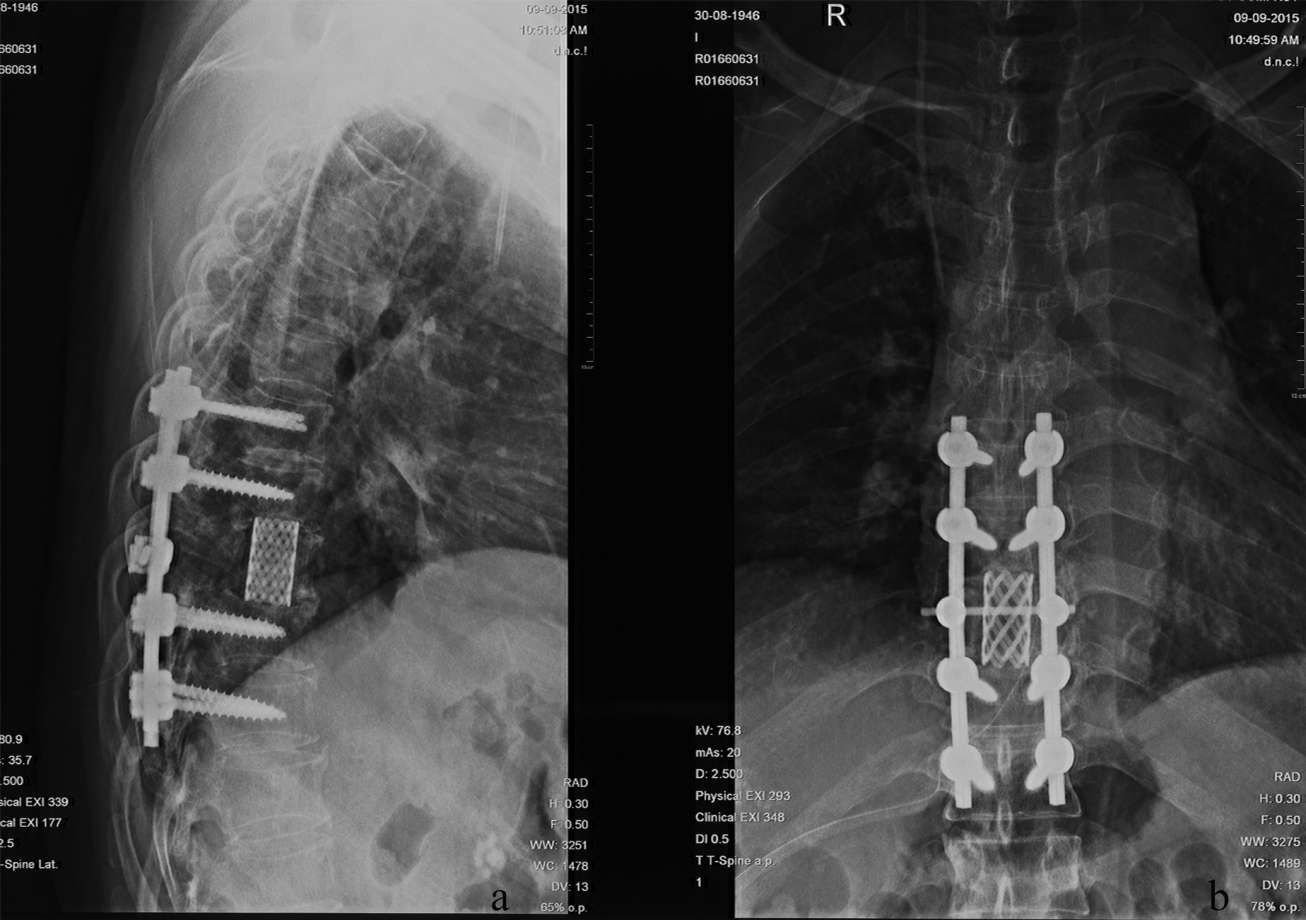
**A**.-**B**. Postoperative X-ray outcome at the final follow-up 59.0 months after surgeries and adjuvant therapies.

HCC is the second leading cause of cancer-related death in China and the third worldwide [24, 25]. Because of improved diagnosis and therapeutic modalities for primary HCC, more cases of extrahepatic metastases, especially bone metastasis, have been detected in recent years. MSCC from primary HCC can be seen as a certain probability in clinical practice and remains a challenge for clinicians. In patients with the terminal stage of advanced cancer associated with persistent pain and neurological defects, surgical intervention could be an effective way to relieve symptoms directly and enhance the quality of life. Figure 3 and 4 illustrate the preoperative radiologic findings and postoperative condition (54 months after spine surgery) of a representative patient who ever underwent excision of primary HCC and metastatic lesion. And he experienced PFS during a final follow-up of 59.0 months, with a satisfactory quality of life after surgery and adjuvant therapies.

To the best of our knowledge, few studies have reported PFS in patients with HCC-related MSCC. In this study, a robust association was found between the surgical procedures used and PFS (*p* = 0.015) in MSCC patients. Beneficial effects on surgical interventions for MSCCs had been confirmed in a randomized trial [26], a meta-analysis [17] and other studies [27, 28]. It was found in our study patients with circumferential decompression might be able to achieve a better functional outcome and progression-free survival rate than laminectomy. Previously studies [26, 29, 30] also reported that laminectomy could not achieve functional improvement directly and completely, and did not show any difference in PFS outcome as compared with non-surgical therapies. However, the impact might be due to the less tumor burden and better surgical indications of enrolled patients in circumferential decompression group than those in laminectomy group. The median OS of MSCC patients in our series was slightly longer than that in other published reports [31–34]. This might be also attributed to better general condition of the patients in our series and the use of personalized adjuvant therapies after surgery.

Multivariate analysis shows that evaluation on the basis of postoperative Frankel Score (*p* < 0.001) might be more appropriate than preoperative Frankel Score for predicting PFS and OS, suggesting that the postoperative Frankel Score may better reflects the true status of the patient's neurological function. ECOG-PS was also found to be a possible independent variable in our study. ECOG-PS is a standard criterion to comprehensively measure and evaluate the living ability of a patient. Relevant studies have also demonstrated that ECOG-PS in MSCC is a possible independent factor from non-small-cell lung cancer [35] and patients with other spinal metastases [22]. Visceral metastasis have been reported as a fatal factor with a long-survival rate below 5% [36]. It was also found to be strongly associated with poor PFS (*p* = 0.011) and OS (*p* < 0.001) in our study, but no correlation was found between other bone metastases and PFS (*p* = 0.156) or OS (*p* = 0.738). The possible reason might be as follows: First, the number of patients involved with other bone metastasis was 40(25.8%), and its impact might be covered by visceral metastases. Second, patients with simple bone metastasis from primary HCC may survive longer than those with visceral metastasis, because bone metastasis itself doesn't result in death directly. We found that the emergence of paravetebral soft tissue neoplasm on MRI had no significant correlation with poor PFS (*p* = 0.192) or OS (*p* = 0.200) in our study. Nonetheless, as long as the tumor exists, there may be an increased possibility for residual cancer cells to survive under radiation [14, 37], which is likely to pose a poor impact on the prognosis.

Radiotherapy (means stereotactic radiation in our study) is a remarkable modality to relieve pain and obtain neurological-deficit-free survival for patients with MSCC [37–39]. A robust association between radiation and PFS (*p* = 0.015) as well as OS (*p* < 0.001) was found in our study. The recent study [34] also showed stereotactic radiation was superior to conventional radiotherapy in the treatment of HCC spinal metastasis. Previous studies [17, 26] advocated that radiotherapy should be performed after direct decompressive surgery, believing that they were superior to radiation alone. Certainly, more high-quality prospective randomized cohort study ought to be performed to validate this potential factor. Sorafenib was found to be another independent prognostic indicator both for PFS (*p* = 0.006) and OS (*p* = 0.024). Cheng et al [40] and Llovet et al [41] reported that Sorafenib was an ideal option for the treatment of advanced HCCs in different large clinical trials. Another latest prospective multicenter cohort study demonstrated that Sorafenib was also appropriate for patients with advanced HCC with extrahepatic metastasis [42]. Despite the favorable outcomes with Sorafenib reported in these studies, its adverse effects and recurrence after drug withdrawing should not be neglected [43, 44]. Systemic chemotherapy (not including TACE or Sorafenib) after surgery does not appear to have a significant correlation with good prognosis. Indeed, whether systemic chemotherapy is effective and safe for MSCC from primary HCC remains controversial [45]. Besides, even though no significant association was found between bisphosphonate treatment and PFS (*p* = 0.359) or OS (*p* = 0.484) in our study, a published experimental study proved that bisphosphonate could prevent the proliferation and migration of HCC cells [46]. Knowing that bisphosphonate can definitely prevent or prolong the emergence of skeletal-related events to help improve the quality of life for patients with bone metastasis [47]. We recommend that it should be administrated routinely after surgery in MSCC patients. Although TACE is regarded as an alternative therapy of surgical resection for advanced HCCs, it didn't seem to prolong PFS or OS as shown in our study. A recent relevant study [48] also suggested that TACE didn't improve the efficacy of treatment for advanced HCCs, and therefore it was not recommended for use in combination with other therapies [49]. In our study, we found that patients who underwent hepatecotomy with or without liver transplantation tended to have a better OS (*p* = 0.006). Surgery remains the optimal treatment option for primary HCC, and liver transplantation should be considered in any patient with a small HCC [50]. Indeed, OS is more relevant to the status of primary tumors instead of metastatic lesions. Therefore, the effective disposal of primary HCC is associated with better prognosis. Liver transplantation has been reported to help gain an excellent long-term survival for early-stage HCC [51]. A recent study [52] also declared that it was a safe and effective option with promising outcomes.

### Limitations

Although this is a novel and the largest sample to date, it has some limitations indeed. First this was a retrospective study and therefore recall bias could not be overlooked. In addition, as the number of involved patients is not large enough, some potential independent factors may have been missed.

In conclusion, surgical interventions could be an alternative treatment for MSCCs caused by primary HCC. Circumferential decompression was associated with better PFS than laminectomy. The postoperative Frankel Score E, ECOG-PS of 1 or 2, no visceral metastasis, administration of postoperative radiation and the use of Sorafenib were found to be significant predictors for better PFS and OS. Patients who previously underwent resection of primary HCC with/without liver transplantation tended to have a better OS.

## Abbreviations

PFS: Progression-free survival
OS: overall survival
MSCC: Metastatic spinal cord compression
HCC: hepatocellular carcinoma
HBV: hepatitis B virus
HCV: hepatitis C virus
PET-CT: Positron emission tomography-computed tomography
ECOG-PS: The Eastern Cooperative Oncology Group performance score
TACE: trans-catheter arterial chemotherapy and embolization
HR: Hazard Ratio
CI: confidence interval
